# Effects of behavioral performance, intrinsic reward value, and context stability on the formation of a higher-order nutrition habit: an intensive longitudinal diary study

**DOI:** 10.1186/s12966-022-01343-8

**Published:** 2022-08-12

**Authors:** Michael Kilb, Sarah Labudek

**Affiliations:** 1 Department of Psychology, School of Social Sciences, University of Mannheim, L13, 17, 68161 Mannheim, Germany; 2 Department of Child Nutrition, Max Rubner-Institut, Federal Research Institute of Nutrition and Food, Haid-und-Neu-Straße 9, 76131 Karlsruhe, Germany; 3 Network Aging Research, Heidelberg University, Bergheimer Straße 20, 69115 Heidelberg, Germany

**Keywords:** Habit strength, Behavioral automaticity, Habit formation, Vegetable intake, Nutrition, Context stability, Perceived reward, Health behavior intervention

## Abstract

**Background:**

Habits drive many of our health behaviors in our daily lives. However, little is known about the relative contribution of different key factors for habit formation in real-world contexts. We examined the effects of behavioral performance, intrinsic reward value (operationalized as tastiness), and context stability on the formation of a higher-order nutrition habit.

**Methods:**

Participants were recruited via mailing lists and posts on social media platforms. *N* = 199 participants (*M*_*age*_ = 37.10 years, *SD* = 13.00, 86.93% female) received an online intervention for building the higher-order habit of *filling half of their plates with vegetables at dinner* and completed one daily online survey for up to 56 days, including the assessment of habit strength, behavioral performance, intrinsic reward value, and context stability, providing a total of *N* = 6352 daily measurements. *N* = 189 participants (*N* = 4175 measurements) could be included in the primary analysis. Utilizing multilevel modeling, we analyzed the impact of behavioral performance, intrinsic reward value, and context stability, as well as their interaction effects, on habit strength on the next day.

**Results:**

Habit strength significantly increased over time. This effect was strengthened in persons with high mean levels of behavioral performance. Furthermore, mean levels of behavioral performance, intrinsic reward value, and context stability were all positively related to mean levels of habit strength. There were no positive effects of daily intraindividual variations in the three examined factors on habit strength at the next day. There was an unexpected negative effect of daily behavioral performance on habit strength at the next day. We found little to no evidence for our expected and pre-registered interaction effects. In an additional exploratory analysis, there were positive effects of daily intraindividual variations in the three factors on habit strength at the same day.

**Conclusions:**

We found that behavioral performance, intrinsic reward value, and context stability were all independent predictors of habit strength of a higher-order habit at the between-person level. However, we did not find the expected associations at the within-person level. Habit interventions should promote the consistent performance of the target behaviors in stable contexts.

**Trial registration:**

https://aspredicted.org/blind.php?x=vu2cg4. Registered 28.04.2020.

**Supplementary Information:**

The online version contains supplementary material available at 10.1186/s12966-022-01343-8.

## Background

A healthy diet, especially regular and sufficient vegetable consumption, is associated with health benefits due to its preventive effect for non-communicable diseases [1, 2]. Population levels of vegetable consumption, however, often lack behind the recommended amount [3] of three portions per day. Effective interventions are needed to increase vegetable consumption in the population long-term.

Habits have been shown to be an important predictor for nutrition behavior [4–6] and habit formation is a promising intervention strategy to improve nutrition [7]. By definition, a habit is a process in which a certain action is promoted automatically by contextual cues due to a mental association between the cue and the behavior which has been formed by frequent cue-dependent repetition of the behavior [8, 9]. When a habit is established, the mere exposure to the contextual cue is assumed to automatically trigger the urge to enact the habitual response [8, 10]. The value of establishing habits for vegetable consumption is that, once established, habits function with little or no cognitive effort and independent of motivational states [11–13] and thereby support behavioral maintenance. The degree to which a behavior is habitual is referred to as *habit strength* [14]. Evidence from empirical studies suggests that higher levels of habit strength regarding healthy eating are associated with higher levels of behavioral frequency and better health outcomes [4, 15]. For example, a recently published intervention study on the effects of a web-based randomized controlled trial (RCT) aiming to improve dietary quality found that increases in habit strength also predicted increases in healthy eating [16].

### Higher-order nutrition habits

Vegetable consumption as a target behavior for habit-based interventions can be considered as a complex reoccurring multi-step health behavior [17], as it needs to be enacted regularly, takes up time resources (doing grocery shopping, preparing vegetables), and can be performed in various situations (e.g., at lunch, dinner, or snack time) and to different extents (e.g., having a burger with one slice of tomato on it vs. eating a salad). Complex behaviors (i.e., eating vegetables) can be habitually *instigated* (a higher-order behavioral target gets activated by the process of habit) and/or habitually *executed* (lower-level actions that serve the higher-order behavioral target get activated by the process of habit) [18]. Even when performed in the same reoccurring context (e.g., when eating dinner), which is a precondition for habit formation, eating vegetables is still a complex behavior which requires several steps and can be either instigated and/or executed habitually [18, 19]. Encountering dinner time could, for example, habitually activate the higher-order behavioral target of eating vegetables at dinner (habitual instigation), but the specific steps performed to achieve this goal could be more conscious and dependent on resources and other factors (non-habitual execution; for example, a person might consciously decide to have a salad for dinner). Eating a salad further involves multiple ‘lower-level’ actions [20] such as cutting the vegetables, preparing the dressing, and eating the salad, which again may or may not be determined by habit (habitual execution).

Instead of forming the habit of eating one specific type and amount of vegetables (i.e., a hand full of cherry tomatoes) in a specific context (when eating dinner), a person could form the habit of eating any vegetables with dinner (i.e., the *instigation* of eating vegetables is tied to a contextual cue) which allows more variety for the actual behavioral execution to achieve the behavioral goal. Forming a *higher-order habit* on vegetable consumption (referring to habitual instigation of eating vegetables), for example filling half of the plate with vegetables, might be a practical method to ensure sufficient and diverse vegetable consumption [21]. This target habit is higher in the behavioral hierarchy than more specific dietary behaviors that have been studied previously [22]. One experimental study showed that participants who formed this higher-order habit reported significantly higher levels of habit strength one, two and three weeks after a habit formation intervention, compared to a control group [21].

### The habit formation process

To increase the strength of a certain habit, the specific behavior must be repeatedly performed in the same context until a mental association between the context and the behavior is established [23]. Habit strength typically increases in the shape of an asymptotic curve with fast increases in the beginning, peak values being reached after around two months, and with large variability between individuals [24, 25]. Intensive longitudinal designs can help to disentangle differential effects in the habit formation process [24, 26, 27].

#### Determinants of habit formation

Gardner and Lally [28, 29] have proposed a framework for habit formation, which includes four stages: Intention formation (stage 1), action initiation (stage 2), behavioral repetition (stage 3a), and development of a cue-behavior-association (stage 3b). Each of these stages can be a potential starting point for examining and modifying factors which may promote successful habit formation. For example, goal setting as a behavior change technique (BCT; BCT 1.4.) [30] could be used to support intention formation (stage 1), whereas action planning (BCT 1.4.) in the form of implementation intentions [31] could be used to promote action initiation (stage 2) and behavioral repetition (stage 3a). Behavioral repetition could be further supported by self-monitoring of behavior (BCT 2.3.), and the development of cue-behavior-associations could be quickened by using rewards (BCT 10.3.), see also [9].

In this study, we simultaneously examine the independent influence of behavioral performance, intrinsic reward value related to vegetable consumption, and the stability of the context in which the target behavior is enacted, on the formation of a higher-order nutrition habit. All three factors have been discussed on a theoretical level [8, 23] and researched within the frameworks of different health behaviors, but mainly physical activity, and mainly on the between-person level (i.e., by examining the influence of the factors on interindividual differences in habit formation over time) [21, 32–37]. With regard to dietary behavior, positive associations between *consistent behavioral performance* and habit strength [21, 25, 38, 39] and mixed associations between *intrinsic reward value* and habit strength exist [21, 32, 39]. Repeated behavioral performance clearly relates to stage 3a and 3b of the habit formation framework [28] and should promote successful habit formation. The intrinsic reward value related to vegetable consumption could potentially enhance motivation or performance and accelerate cue-behavior learning and therefore positively affect multiple stages of the habit formation process [28, 40]. With regard to the third factor, context stability, some studies have investigated which kind of cues (e.g., event-based vs. time-based cues) promote habit formation best [24, 41]. However, there is no research on the influence of the *stability of contextual cues* on the formation of real-world nutrition habits [36]. Regarding vegetable consumption at dinnertime, there are various contextual factors such as the time and position in the daily routine (e.g., after work or exercising), location, social context (i.e., how many other persons were present), and the type of vegetables consumed.

Taken together, it can be assumed that more frequent behavioral performance, higher intrinsic rewards experienced, and higher context stability are positively associated with habit strength and increases of habit strength over time (when examining *inter*individual differences between individuals). In contrast, only little empirical research has been conducted on the effects of *intra*individual variations in the three different factors on habit strength (e.g., the effect of higher than usual experienced intrinsic reward at one day on habit strength at the next day) [39], which warrants further investigation. There is also little information on the temporal resolution of factors influencing habit formation, because only few intensive longitudinal habit formation studies exist, and lagged effects are rarely tested (see [39] for a recently published exception). As habit strength can be considered as the cached value of previous repetitions, performing the behavior on one day can be expected to lead to higher habit strength at the next day. Furthermore, because *context-dependent* repetition of the target behavior is essential for establishing cue-behavior associations [23, 29], a higher cue or context stability should increase the effect of performing the target behavior on habit strength. Finally, experienced rewards in timely proximity to behavioral performance are also expected to positively modulate and increase the effect of repeated behavioral performance [8]. The relative importance – when considered simultaneously – and the moderating effects of intrinsic reward value and context stability on the effect of behavioral performance at both the between- and within-person level remain unclear, as the three factors rarely get examined simultaneously.

### Aim of the study

Following design and measurement guidelines for tracking real-world habit formation [27], this study examines both between- (person level) and within-person (day level) effects of behavioral performance, intrinsic reward value, and context stability as determinants of habit formation of a higher-order habit of *‘filling half the plate with vegetables at dinner time’* [21] following a short habit intervention in an intensive longitudinal design. With this study, we adhere to the guidelines for rigorous habit formation research [42]. Additional file 1 contains the methodological characteristics of this study against the criteria proposed by Gardner et al. [42].

We expect 1) a positive effect of time since the intervention on habit strength at the within-person level, that is, behavioral automaticity will increase over time; 2) positive between-person effects of consistent behavioral performance, context stability, and intrinsic reward value as well as positive two-way interaction effects of these predictors with time on habit strength; 3) positive effects of behavioral performance, context stability, and intrinsic reward value as well as the two-way interactions with behavioral performance on habit strength at the within-person level.

## Methods

This study was pre-registered (https://aspredicted.org/blind.php?x=vu2cg4) and approved by the Institutional Review Board of Heidelberg University (AZ Labu 2020 1/ 1) prior to data collection. Please see Additional file 2 for the completed STROBE checklist for cross-sectional studies.

### Participants

**Fig. 1:**
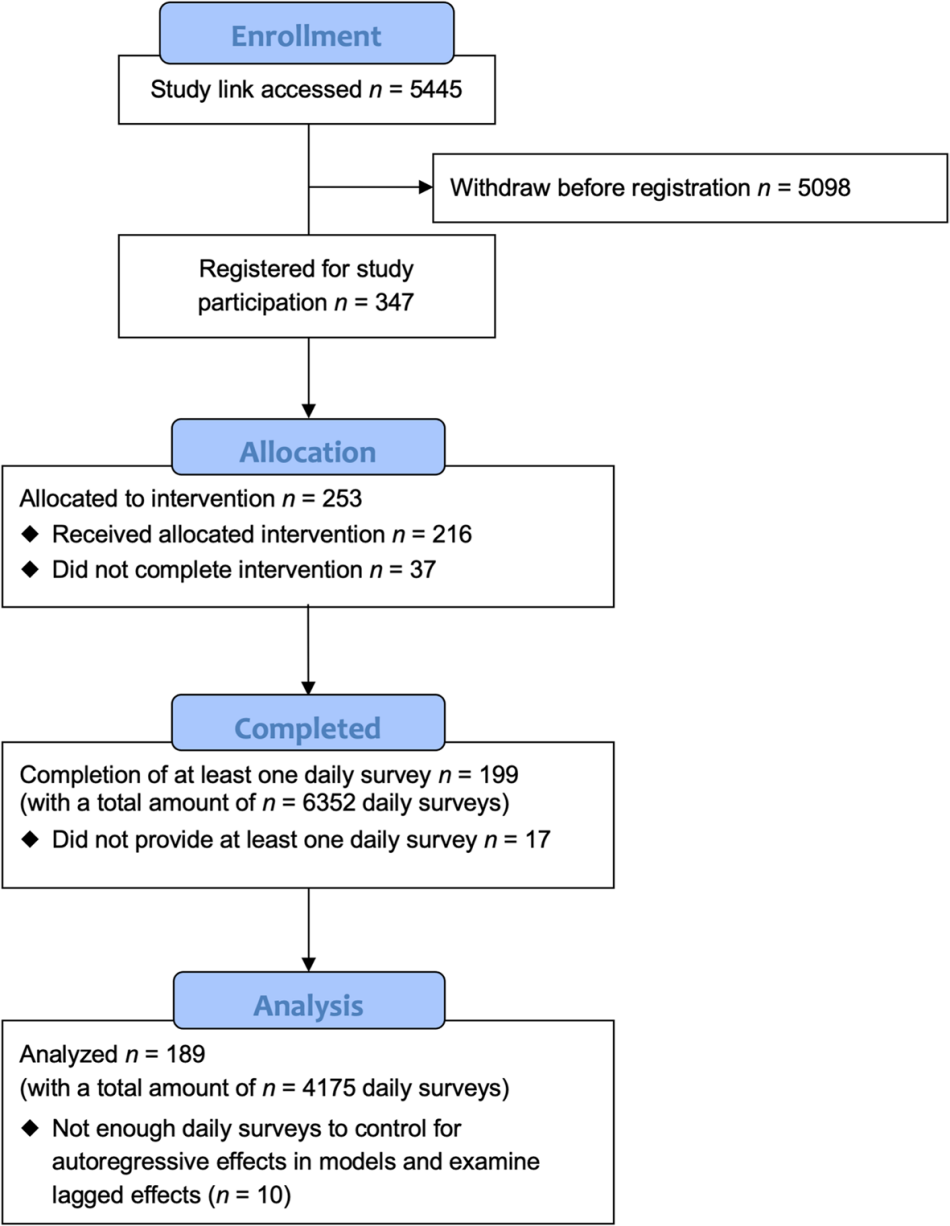
Participant flow chart

We aimed to recruit about 80–90 participants with about 3570 data points (85 participants on level 2 * 56 daily surveys on level 1) to reliably estimate small fixed effects at the day-level, in case of small ICCs, as well as at least medium sized cross-level interaction effects with medium slope variances [43, 44]. In total, *N* = 199 participants (level 2) completed at least one daily survey and provided overall *n* = 6352 daily surveys (level 1). *N* = 189 participants could be included in the final analyses because they provided enough daily diaries for estimating the specified autoregressive effects. Please see Fig. 1 for the participant flow chart.

### Procedure

Participants were recruited via mailing lists, posts on social media platforms, and paid advertisement via social media platforms. Data collection for the baseline assessment took place between July 2020 and January 2021. Participants were eligible if they were at least 18 years old, had access to a computer or mobile device and email, as well as a WhatsApp account. Participants had the opportunity to enter a lottery to win one out of ten online shopping vouchers (worth 50€ each). The winning probability was linked to participants’ study participation, in the form that the winning probability increased with every completed survey and photo documentation of their dinner. After registration and provision of informed consent, participants received a link to the baseline assessment where participants provided demographic information, information about control variables, as well as variables of interest. Subsequently, they completed a single session habit formation intervention (see below). The diary period started the day after participants completed the baseline assessment; participants received one daily survey for eight weeks (i.e., 56 days) at 8.30 p.m. and were asked to fill out the daily survey after having eaten dinner. Completion of the survey was possible until 11 p.m.

### Habit formation intervention

Participants were supported in building the higher-order habit of filling half of their plate with vegetables at dinner [21]. In a first step, benefits of regular vegetable consumption were displayed (BCT 5.1. Information about health consequences) and information on current recommendations for vegetable consumption and portion sizes was provided (BCT 4.1. Instruction on how to perform the behavior). Participants subsequently received information on habits and their benefits for health behavior maintenance and were familiarized with the target habit. Participants were then educated about important factors in the habit formation process and how to use them for habit formation (BCT 4.1. Instruction of how to perform the behavior), for example, context-dependent repetition of the target behavior (BCT 8.3. Habit formation). Participants then chose a contextual cue for their habit (BCT 7.1. Cues/prompts; 12.1. Restructuring the physical environment). Furthermore, participants learned that rewards associated with the behavioral performance can promote habit formation and how this might be applied to the study context (e.g., preparing food in a tasty manner and serving it nicely). A list of webpages with vegetable-based recipes was provided. Afterwards, participants were asked to form implementation intentions [31] with their prior specified contextual cue (BCT 1.4. Action planning). Likewise, participants were asked to specify potential barriers for the performance of the target behavior and provide coping plans on how to overcome the barriers (BCT 1.2. Problem solving; BCT 1.4. Action planning). They were also asked to send a picture of their dinner plate to a study WhatsApp account for the whole habit formation and diary period (BCT 2.3. Self-monitoring of behavior). Participants received an email with a summary of their provided information after completing the intervention. Please see Additional file 3 for the completed TIDieR checklist.

### Measures

#### Baseline assessment

*Demographic variables* of age (years), weight (kg), height (cm), current state of employment, and highest educational attainment were assessed at baseline. Educational level was coded according to the International Standard Classification of Education (ISCED) [45] and the recommendations by Eurostat [46] into the levels low (up to completing lower secondary education; ISCED levels 0–2), medium (completing higher secondary education and post-secondary, non-tertiary education; ISCED levels 3–4) and high (from completing the first stage of tertiary education up to completing doctoral level; ISCED levels 5–8), which allows cross-country comparisons [46].

*Nutrition preferences* (e.g., omnivore, vegetarian, vegan) were assessed with the self-generated item “What most closely reflects your current diet?”. Participants could choose one out of six options (omnivore, vegetarian, vegan, pescatarian, paleolithic, or other).

*Food intolerances* were assessed with the self-generated item “Do you have any food intolerances?” (no = 0, yes = 1).

*Average vegetable intake at dinner* and *average daily vegetable intake* was assessed in 0.25-portion steps with two adapted dropdown items [47]; example item: ‘Please indicate how many servings of vegetables you have consumed per day, on average, over the past seven days’ after receiving information about portion sizes, based on the recommendation of the German Federal Ministry of Nutrition and the German Five-A-Day Campaign [48, 49].

*Health consciousness* was assessed with five items [50] on a 5-point Likert-scale (1 = ‘do not agree at all’ to 5 = ‘totally agree’); example item: “Living life in best possible health is very important to me”; Cronbach α = 78. For the analyses, the mean value of the five items was used.

The *weekly frequency of eating dinner* was assessed with the self-generated item “How many days per week do you eat dinner?” on an 8-point Likert-scale (0 = ‘0 days’ to 7 = ‘7 days’).

The *context stability of the dinner* (stability of dinner time, location, social environment, types of vegetables, and activities prior to and post dinner) was assessed with six items on an adapted scale by Pimm et al. (2016) on a visual analogue scale (0% = ‘always changing’ to 100% = ‘always the same’); example item: “How consistent is the location at which you eat dinner?”; Cronbach α = 74. For the analyses, the mean value of the six items was used and divided by 100.

#### Daily survey

*Habit strength* was assessed with the Self-Report Behavioral Automaticity Index [14]. After the prefix “filling half of the plate with vegetables at dinner time is something…”, participants were asked to rate four statements on behavioral automaticity, e.g., “…I do automatically” on a 7-point Likert-scale (1 = ‘do not agree at all’ to 7 = ‘totally agree’). For the analyses, the mean value of the four items was used. Internal consistency, assessed with Nested Alpha for multilevel data [51] was α = 0.99.

*Behavioral performance* was assessed with the dichotomous item “Did you fill half of your plate with vegetables at dinner?” (0 = no, 1 = yes).

The *intrinsic reward value* of dinner was assessed via two self-generated items with a 5-point Likert-scale (1 = ‘do not agree at all’ to 5 = ‘totally agree’) regarding the tastiness of the dinner; example item: “The vegetables at dinner were tasty”; internal consistency, assessed with Nested Alpha for multilevel data [51], was α = 1.00.

*Context stability* of dinner (regarding time, location, social environment, types of vegetables and activities prior to and post dinner being “as usual”) was assessed via six adapted items [33] on a 7-point Likert-scale (1 = ‘do not agree at all’ to 7 = ‘totally agree’); example item: “Dinner tonight took place in the same location as usual.”. For the analyses, the mean value of the six items was used. Internal consistency, assessed with Nested Alpha for multilevel data [51], was α = 0.94.

### Data analysis

Data analysis was performed using R version 4.0.3. The R scripts containing the main analyses are available on https://osf.io/z9f4v/.

To examine the simultaneous influence of behavioral performance, context stability, and intrinsic reward value on habit strength at the next day, as well as habit formation over time, we specified two multilevel models using the R-package lmerTest [52]. Due to the robustness of multilevel models to deviations from normal distribution [53], we did not alter primary data in any way. In all multilevel models, daily diaries (level 1) were nested within individuals (level 2). For examining between-person effects, values of the predictors represented the individual person-mean [54]. For within-person analyses, predictors were centered within individuals such that positive values of predictors represented higher than average values, compared to the individual person-means, i.e., daily deviations from the person mean [54]. In the whole manuscript, we report the standardized effects.

We conducted the pre-registered analyses with all participants (model 1a) as well as all participants who responded to more than 50% of the daily surveys (high compliance sample; model 1b) to test the robustness of the results. The models included a random intercept for persons, person-centered values and person-specific means of behavioral performance, context stability, and intrinsic reward value at t1, and an autoregressive effect of habit strength (i.e., habit strength at t1) as independent variables. As dependent variables, habit strength at t1 + 1 were analyzed. All models included time and log(time) at t1 as independent variables to take the asymptotic nature of the habit formation curve into account [24, 25]. We specified random slopes for log(time) to model the between-person variability in habit formation over time and to examine cross-level interactions. The models further included the following interaction effects: 1) cross-level interactions of log(time) with person-specific means of behavioral performance, context stability, and intrinsic reward value to predict between-person variability in habit formation over time and 2) interactions of person-centered behavioral performance with person-centered context stability and intrinsic reward value. Additionally, we also exploratorily estimated the comparable within-person effects on habit strength at the same day (models 2a and 2b; Additional file 4).

The final analyses deviated from the pre-registration in the following ways: First, between- and within-level effects of behavioral performance, context stability, and intrinsic reward value were included in the same model instead of estimating separate models. As person-mean centering disentangles between- and within-person variance [54], both effect types can be examined in one shared model. Second, in all models, we also included an autoregressive effect, i.e., habit strength of day x-1 was included as a predictor to control for potential autocorrelation [55]. Third, we also included a log-transformed time predictor to account for the asymptomatic nature of the habit formation process [29]. The interaction effects of mean behavioral performance, context stability, and intrinsic reward value with time were thus estimated with the log-transformed time predictor instead of the linear time predictor. Finally, we conducted an additional exploratory analysis to examine the comparable within-person effects (on habit strength at the same day as dependent variable) at a higher temporal resolution.

## Results

### Sample characteristics

Of the *N* = 253 of participants who completed the baseline assessment, *n* = 199 responded to one or more daily surveys (Fig. 1). The final sample (*N* = 199) comprised of 86.93% females (9.55% male, 3.52% missing values). On average, participants were 37.10 years old (*SD* = 13.00 years, range 18.00 – 70.00 years). Most participants (78.39%) were employed. Regarding education, 24.12% reported a low level, 44.72% a medium level, and 31.16% a high level. Please see Table 1 for a detailed overview of all baseline characteristics.

**Table 1:** Baseline characteristics of all participants and by respondence status

Variable	All (*N* = 253)	Non-responder (*n* = 54)	Responder (*n* = 199)	*p*
Sex (female), *n* (%)	220 (86.96)	47 (87.04)	173 (86.93)	.388
NA	7 (2.77)	0 (0.00)	7 (3.52)	
Age (years), *M* (*SD*)	36.90 (12.99)	36.20 (13.05)	37.10 (13.00)	.657
Educational level, *n* (%)				.089
Low (ISCED levels 0–2)	69 (27.27)	21 (38.89)	48 (24.12)	
Medium (ISCED levels 3–4)	110 (43.48)	21 (38.89)	89 (44.72)	
High (ISCED levels 5–8)	74 (29.25)	12 (22.22)	62 (31.16)	
NA	0 (0.00)	0 (0.00)	0 (0.00)	
Employment, *n* (%)				.150
Not employed	60 (23.72)	17 (31.48)	43 (21.61)	
Employed	193 (76.28)	37 (68.52)	156 (78.39)	
BMI (kg/m^2), *M* (*SD*)	27.70 (6.70)	28.23 (7.99)	27.56 (6.30)	.580
Food intolerance (yes), *n* (%)	48 (18.97)	9 (16.67)	39 (19.60)	.273
NA	1 (0.40)	1 (1.85)	0 (0.00)	
Diet				.282
Omnivore	168 (66.40)	40 (74.07)	128 (64.32)	
Vegan	8 (3.16)	2 (3.70)	6 (3.02)	
Vegetarian	33 (13.04)	7 (12.96)	26 (13.07)	
Pescetarian	18 (7.11)	2 (3.70)	16 (8.04)	
Paleo diet	2 (0.79)	0 (0.00)	2 (1.01)	
Other	23 (9.09)	2 (3.70)	21 (10.55)	
NA	1 (0.40)	1 (1.85)	0 (0.00)	
Frequency of dinner per week, *M* (*SD*)	6.43 (1.39)	6.15 (1.65)	6.50 (1.30)	.154
Health consciousness, *M* (*SD*)	3.70 (0.64)	3.53 (0.62)	3.75 (0.63)	.029
Context stability, *M* (*SD*)	0.63 (0.18)	0.61 (0.20)	0.63 (0.17)	.454
Daily vegetable portions, *M* (*SD*)	1.68 (1.31)	1.73 (1.75)	1.67 (1.17)	.822
Daily fruit portions, *M* (*SD*)	1.34 (1.42)	1.75 (2.06)	1.24 (1.20)	.090

*Note*. Participants were categorized as responders when they answered at least one daily survey. NA = missing values. BMI = body mass index (kg/m^2). ISCED = International Standard Classification of Education [45]. Educational level was aggregated according to recommendations by Eurostat [46]

### Descriptive statistics

Participants completed on average *M* = 31.92 daily surveys (*SD* = 18.49) which represents 57.00% of the maximum of 56 daily surveys. In total, 58.79% of the sample responded to more than 28 daily surveys (50%). Mean values, standard deviations, and ICCs of the predictor and outcome variables are displayed in Table 2.

**Table 2:** Descriptive statistics of the predictor and outcome variables

	***M* (*SD*)**	**ICC**
Behavioral performance	0.70 (0.46)	0.12
Intrinsic reward value	4.61 (0.70)	0.27
Context stability	5.07 (1.32)	0.33
Habit strength	4.00 (1.86)	0.69

*Note.* ICC = Intraclass coefficient. ICC values can range from 0 to 1, indicating the ratio of the between-cluster (i.e., between-person in this case) variance to the total variance. For example, the ICC of 0.69 in habit strength indicates that 69% of the variance in habit strength is due to differences between individuals. Higher values can be seen as an indicator for the necessity of using multilevel modeling

### Model 1a and 1b: Time-lagged effects of daily variations in behavioral performance, context stability, and intrinsic reward value on habit strength at the next day

Standardized fixed effects of all predictors and control variables, random effects and 95% confidence intervals of the analyses with all participants (model 1a) and participants with high compliance (model 1b) are displayed in Table 3. There were positive main effects of time and log(time) on habit strength in both analyses (see Table 3).

**Table 3:** Results of the next-day multilevel models for the outcome habit strength with all participants (model 1a) and participants with high compliance (model 1b)

Predictors	Model 1a				Model 1b			
	**β**	***SE***	***95% CI***	***p***	**β**	***SE***	***95% CI***	***p***
Intercept	-0.01	0.04	[-0.08, 0.06]	.798	0.00	0.05	[-0.09, 0.09]	0.974
**Within-person effects**								
Autoregressive effect	0.32	0.02	[0.28, 0.35]	**< .001**	0.28	0.02	[0.24, 0.32]	**< .001**
Time	0.05	0.02	[0.02, 0.09]	**.002**	0.05	0.02	[0.02, 0.09]	**.001**
log(time)	0.06	0.02	[0.02, 0.10]	**.006**	0.06	0.02	[0.01, 0.10]	**.010**
Behavioral performance (cwc)	-0.03	0.01	[-0.04, -0.01]	**< .001**	-0.03	0.01	[-0.04, -0.01]	**.001**
Intrinsic reward value (cwc)	-0.00	0.01	[-0.01, 0.01]	.932	0.00	0.01	[-0.01, 0.02]	.861
Context stability (cwc)	0.01	0.01	[-0.01, 0.02]	.401	0.01	0.01	[-0.01, 0.02]	.370
Behavioral performance (cwc)*Intrinsic reward value (cwc)	0.00	0.01	[-0.01, 0.01]	.608	0.00	0.01	[-0.01, 0.01]	.673
Behavioral performance (cwc)*Context stability (cwc)	-0.00	0.01	[-0.02, 0.01]	.693	-0.01	0.01	[-0.02, 0.01]	.391
**Between-person effects**								
Behavioral performance (M)	0.16	0.03	[0.09, 0.22]	**< .001**	0.11	0.05	[0.02, 0.20]	**.015**
Intrinsic reward value (M)	0.08	0.04	[0.01, 0.15]	**.036**	0.15	0.05	[0.05, 0.25]	**.003**
Context stability (M)	0.20	0.04	[0.13, 0.28]	**< .001**	0.23	0.05	[0.14, 0.33]	**< .001**
log(time)*Behavioral performance (M)	0.03	0.01	[0.01, 0.06]	**.015**	0.03	0.02	[0.00, 0.07]	**.033**
log(time)*Intrinsic reward value (M)	0.01	0.01	[-0.02, 0.04]	.459	0.02	0.02	[-0.01, 0.06]	.179
log(time)*Context stability (M)	0.02	0.02	[-0.01, 0.05]	.215	0.02	0.02	[-0.02, 0.05]	.325
**Random effects**								
*SD*(Residual)	0.44	0.01	[0.43, 0.45]		0.42	0.01	[0.41, 0.43]	
*SD*(Intercept)	0.46	0.03	[0.41, 0.51]		0.48	0.03	[0.43, 0.55]	
*SD*(Slope (log(time)))	0.15	0.01	[0.12, 0.17]		0.15	0.01	[0.13, 0.18]	
ICC	0.54				0.59			
* N*	189				121			
Observations	4175				3753			
Marginal R^2^ / Conditional R^2^	0.439 / 0.743				0.439 / 0.770			

*Note.* The next-day models were estimated with habit strength measured one day after the day when predictors were measured. (M) = Person-mean values. (cwc) = Values centered within clusters (persons). ICC = Intra-class correlation. *N* = Number of participants. *p*-values < .050 are marked in bold. Model 1a contains the analysis with all participants that could be included in the analysis. Model 1b contains the analysis with participants who responded to more than 50% of the daily surveys (high compliance sample)

#### Between-person effects

Individuals reporting higher mean behavioral performance (β = 0.16, *p* < .001), higher mean context stability when eating dinner (β = 0.20, *p* < .001), and higher mean perceived intrinsic reward value of the dinner (β = 0.08, *p* = .036) showed on average higher levels of habit strength compared to individuals with lower means of behavioral performance, context stability, and intrinsic reward value (see Table 3). Furthermore, there was a positive statistically significant interaction effect of the person-specific mean behavioral performance and log(time) (β = ﻿0.03, *p* = .015), indicating that habit strength increased more strongly in individuals with higher mean levels of behavioral performance. All other cross-level interaction effects of the between-person predictors and log(time) were statistically non-significant (see Table 3).

#### Within-person effects

Participants reported lower habit strength on days that followed days on which they had performed the target behavior (β = -0.03, *p* < .001). There were no other lagged within-person effects (see Table 3).

#### Analyses with high compliance sample

In the model with the high compliance sample (model 1b), all statistically significant main and interaction effects of model 1a replicated (see Table 3).

### Exploratory analyses: Same-day effects of daily variations in behavioral performance, context stability, and intrinsic reward value on habit strength at the same day

We exploratorily examined the comparable within-person associations between daily variations in behavioral performance, context stability, and intrinsic reward value, with habit strength at the same day to look at potential within-person effects at a higher temporal resolution (i.e., temporal proximity). The detailed results can be found in Additional file 4.

On days on which participants performed the behavior, ate in a relatively more stable context (compared to the personal average), and perceived the consumed dinner as relatively more rewarding (compared to the personal average), levels of habit strength were significantly higher (all β > 0.01, all *p* < .003). Additionally, there was a negative interaction effect of behavioral performance and intrinsic reward value (β = -0.02, *p* < .001), indicating that habit strength was only higher on days when participants rated their dinner to be relatively more tasteful and did *not* fill half of their plate with vegetables (but not when they did). However, all within-person effects were relatively small. In the analyses with the high compliance sample, all within-person effects at the same day replicated.

## Discussion

Utilizing an intensive longitudinal design, we examined both between-person and within-person associations of behavioral performance, intrinsic reward value, and context stability with habit strength in the formation of a new higher-order nutrition habit (filling half of the plate with vegetables at dinner). Our hypotheses could partially be confirmed. First, we found the expected positive effect of time on habit strength. Second, regarding the between-person level effects, we found that mean levels of behavioral performance, intrinsic reward value, and context stability all were independent predictors of mean habit strength levels. Regarding the size of the effects, context stability showed the strongest association, directly followed by behavioral performance, whereas the association of intrinsic of reward value was significantly smaller. However, when including the interaction effects with time, we only found the expected interaction effect with behavioral performance but not with intrinsic reward value or context stability. There were none of the expected within-person effects of daily variations in behavioral performance, intrinsic reward value, and context stability on habit strength at the next day (but there were positive effects in comparable exploratory analyses with habit strength at the same day as dependent variable). In the following, we will discuss the findings of each of our three proposed predictors separately across the between- and within-person level.

Regarding behavioral performance, we found a positive association between behavioral performance and habit strength on the between-person level and an interaction of time and behavioral performance, yielding evidence that consistent behavioral performance is important for habit formation. The results imply that persons with higher mean levels of behavioral performance report higher mean levels of habit strength, and they also show a stronger increase of habit strength over time. This is in line with the findings from earlier studies [21, 24, 25, 32] which found associations between the behavioral performance of the habit and past behavior with habit strength. Contrary to expected, performing the behavior on a given day negatively predicted habit strength at the next day. This contradicts core assumptions of habit theory which proposes that habit strength represents the accumulated number of previous context-dependent behavioral repetitions [23]. However, this assumption might translate into a positive association between behavioral performance and habit strength only at the *between*-person level (i.e., more frequent behavioral performance is reflected in higher habit strength), which we also find in our study, but not at the *within*-person level. Moreover, the negative association *within*-persons could be explained by higher self-regulatory efforts (and thus low experienced automaticity) on days on which participants performed the behavior. Self-regulation is required in the initial and subsequent phase of the habit formation process [29]. The within-person association of behavioral performance and habit strength could be also phase-specific: There might be a negative association in the initial phases of the habit formation process (indicating high self-regulatory efforts and low automaticity) and a positive association in the later phases where the habit has been established to some extent (indicating low self-regulatory efforts and high automaticity).

Regarding intrinsic reward value, participants with higher mean levels of intrinsic reward value showed higher levels of habit strength. This is in line with research indicating that intrinsic exercise rewards predict exercise frequency indirectly via habit strength for exercise maintainers [35]. However, intrinsic reward value did not serve as a moderator on the effect of time on habit strength. On the other hand, foundational research suggests that the strength of the cue-behavior association is modulated by the strength of reward experienced in timely proximity [8]. The missing effect could be at least partially explained by a ceiling effect for intrinsic reward value, as participants rated eating vegetables as quite rewarding (*M* = 4.61 at a maximum value of 5). The results of one study show that the intrinsic reward value of eating fruits and vegetables served both as a mediator and moderator in the relationship between behavior (i.e., fruit and vegetable consumption) and habit strength [32]. The different results might be further explained by different measures of reward value, different study designs, or the lack of controlling for other key factors, as we did in our study. At the within-person level, daily variations in intrinsic reward value did not positively predict habit strength at the next day (i.e., higher than usual experienced intrinsic reward value did not predict habit strength at the next day).

Lastly, our findings also revealed context stability as a consistent predictor of habit strength on the between-person level. More specifically, individuals with higher mean levels of context stability of the situation where the habit takes place (dinner) showed higher mean levels of habit strength. Existing studies show mixed results regarding the effects of context stability on habit strength, which may be due to a stronger differentiation of contexts and cues. For example, there was a positive association of the consistency of some types of cues (e.g., people and mood cue consistency), but not of others (e.g., time and location cue consistency), with physical activity habit [33]. In another study, there were positive associations between the consistency of different types of cues and physical activity behavior, but not physical activity habit [36]. There was no within-person effect of daily variations in context stability on habit strength at the next day. Since the study was conducted during the Covid-19 pandemic, the results might have been different without social contact restrictions. During the study period, participants might have had more stable dinner contexts than outside of the pandemic. Study participants rated the context stability to be quite high in our study (5.07/6). However, the ICC of context stability of 0.33 indicates that the intra-individual variance in context stability was high (67%). That is, dinner contexts seemed to already differ to a great extent within individuals in our study. More studies are needed to examine the influence of larger contextual or cultural influences on individual habit formation.

Exploratorily, we also looked at within-person associations of the three factors with habit strength at the same day to examine potential effects at a higher temporal resolution. In contrast to the lagged-effects analyses, we found that daily variations in behavioral performance, intrinsic reward value, and context stability were positively associated with habit strength at the same day. That is, on days when participants performed the target behavior, where participants found their dinner to be particularly appetizing, and when participants ate in a relatively more stable context, they also reported higher habit strength. Behavioral performance showed the strongest association with habit strength, followed by intrinsic reward value and context stability. However, the effects were relatively small. Surprisingly, there was an unexpected negative interaction effect of behavioral performance and intrinsic reward value, suggesting that the effect of intrinsic reward value on habit strength at the same day was stronger when participants did not execute the target behavior. A potential explanation might be that the taste of dinner promotes habit formation better when participants do not stick to the recommendation of filling half of their plate with vegetables (i.e., not fully execute the target behavior) but rather eat a smaller portion of vegetables. As already discussed, these positive effects were not replicated in the lagged-effects models. Interestingly, in a recently published study, the authors found a comparable results pattern as we did, showing positive effects of intrinsic rewards on habit strength at the between-person level, and also at the within person-level, but only on habit strength at the same day and not the next day [39].

Our results have three important theoretical implications. First, more established research findings regarding the importance of the examined factors (e.g., behavioral performance) for explaining *inter*individual differences in successful habit formation may not necessarily translate to *intra*individual processes and effects (i.e., a positive effect of behavioral performance on habit strength at the next day). This is not an unusual phenomenon as other research also shows opposing associations at the between- and within-person level or even mutual interdependence [56, 57]. Second, the independent positive effects of the three factors at the between-person level suggests that the three factors have from each other independent additive effects on habit strength. Third, the differing results from the next- and same-day analyses might imply that the three factors have a more direct than delayed effect on habit strength. This conclusion is, however, limited by the cross-sectional nature of the same-day analyses, which introduces common method bias and could thereby inflate the observed associations [58].

### Strengths and limitations

This is one of the first studies which tested multiple key factors of habit formation jointly in the context of a newly formed nutrition habit in a non-student sample. The study has several strengths. First, it follows recently formulated design and measurement guidelines for tracking real-world habit formation (25), including an intensive longitudinal design with up to 56 days of measurement per participant. Second, it allows for assessing the unique contribution and interplay of three key factors in the habit formation process. Third, it tests next-day effects, thereby providing more valid indicators for causality and reducing same source bias [59]. Fourth, as former research criticized the missing consideration of within-person variance in individual processes such as habit formation [26], our study provides novel insights into the relevance of different factors at both the between- and within-person level. Lastly, the sample we draw was balanced regarding educational level, which increases the generalizability of the results. Other research suggests that digital interventions might not work equally well for individuals with different socioeconomic status [60].

On the other hand, the present study also has some limitations. First, we integrated the stability of six contextual variables into one measure of context stability. Disentangling which factor shows the strongest association with habit strength would have exceeded the scope of our study. However, recent research suggests there may be differences in the relevance of different cues for habit formation [36]. To provide practical guidelines on habit formation, it would be important to understand which contextual factors are most helpful for habit formation [61]. Second, we examined the formation of a pre-set higher-order habit which might not reflect participants’ own goals or fit to their daily schedules. Although this study was rather about the processes behind habit formation than intervention delivery, it might be that these processes unfold in a different manner when participants receive an intervention which is better tailored to their needs. Third, we used self-report measures which can induce common method bias [58] and lead to biased recall. However, in research on dietary behavior [62] and habit formation [23], subjective measures are frequently used and might be the most appropriate and available tools at a large scale. Individuals often have the most accurate information regarding their behavior, subjective measures are low in cost, and easily applicable in online studies [62]. Furthermore, some measures such as habit strength regarding real-world habits may not be objectively assessable [23]. We aimed to reduce recall bias by assessing the variables of interest in timely proximity to the completion of the target habit. We also partially reduced same source bias, which can exaggerate common method bias, by examining lagged effects [59]. Nevertheless, future research can benefit from using more objective measures where possible such as automatic coding of food pictures to capture behavioral performance [63] or using neurophysiological markers such as heart rate or skin conductance for assessing affective responses to meals [64]. Finally, our study did not include a control group without habit formation intervention and thus does not allow conclusions regarding overall intervention effectiveness. However, as we aimed to examine important moderating factors that might contribute to successful habit formation instead of overall intervention effectiveness, a control group was not necessary.

### Implications for research and practice

Future studies could build on the insights gained from this study. The interval time-lag we used was the closest possible (+ 1 day) in this study but might not reflect the true nature of the within-person effects of behavioral performance, intrinsic reward value, and context stability on habit strength. It is likely that the effects of the three factors on habit strength unfold faster, for example within seconds or minutes after a cue gets associated with the specific behavior, as research suggests that habitual learning shows direct effects on relevant brain regions [8, 65]. Furthermore, research on food reward suggests that different properties of foods such as visual, olfactory, and gustatory cues lead to timely activations of reward-associated brain regions [66, 67]. Therefore, studies with a higher sampling frequency are needed to understand the timely resolution of the effects of behavioral performance, intrinsic reward value, and context stability on habit strength in real-world contexts. Furthermore, habit formation research would generally benefit from using more intensive longitudinal methods and ecological momentary assessment to better understand both, *intra*individual processes in habit formation, and predictors of the *inter*individual variability in trajectories of habit formation. Disentangling between- and within-person effects can help to shed light into potential differences in within- and between-person processes and the temporal dynamics of effects [26, 27, 68].

The fact that our study provides additional proof for habit formation using a higher-order habit could be helpful for future habit-based interventions, especially in complex health behaviors such as nutrition. In real-world settings, higher-order food habit such as filling half of the plate with vegetables at dinner time could promote a high variety in vegetable choice which may improve health outcomes, but findings are mixed [69–72]. In addition, individuals could maintain interest in the behavior over time and be creative and intrinsically motivated in the composition of different types of vegetables. Considering global and national dietary recommendations, food-based dietary guidelines should include specific and actionable advice on the consumption of healthy food groups such as fruits and vegetables. Our results and the results of others [21] show that behavioral targets such as filling half of the plate with fruits and/or vegetables, which is already recommended in some dietary guidelines [73], are suitable for habit formation and thereby the promotion of a healthy diet. Behavioral performance was revealed to be the strongest predictor for habit strength, followed by context stability. Intervention designers and practitioners should thus focus on fostering the consistent performance of the target behavior – ideally in stable reoccurring contexts – for establishing strong healthy eating habits. To ensure context-dependent repetition, additional self-regulatory strategies may further support habit formation [29]. For example, planning with implementation intentions, or self-monitoring, which we included in our intervention, are frequently utilized behavior change techniques in habit-based intervention [9]. Furthermore, other additional behavior change techniques can support habit formation in each of the three phases of habit formation [28, 29]. For example, increasing the intrinsic reward value by making the target behavior more enjoyable [74] could further strengthen habit formation.

## Conclusions

This study examined the effects of behavioral performance, intrinsic reward value, and context stability on habit strength of a higher-order nutrition habit following a short habit formation intervention. Behavioral performance, intrinsic reward value, and context stability did not predict habit strength at the next day (but independently predicted habit strength at the same day). Future studies could investigate whether time-lag intervals other than one day better capture the effects of behavioral performance, intrinsic reward value, and context stability on habit strength, and disentangle which contextual stability determinants (cues) are most influential. Consistent behavioral performance and context stability further emerged as key predictors of habit strength on the between-person level. Thus, in practice, intervention designers should focus on supporting individuals to perform the target habit frequently in a stable context by combining different behavior change techniques that might promote these factors (e.g., implementation intentions for increasing behavioral performance).

## Supplementary Information

**Additional file 1. **Comparison of methodological characteristics of this study with the guidelines for tracking real-world habit formation by Gardner et al. (2022).

**Additional file 2. **STROBE checklist. Checklist which references to the pages in the manuscript which contain the essential descriptions of the study.

**Additional file 3. **TIDieR checklist. Checklist which references to the pages in the manuscript which contain key information on the habit-formation intervention.

**Additional file 4. **Exploratory analysis of same-day associations.

## Data Availability

The dataset supporting the conclusions of this article is available in the Open Science Framework repository, https://osf.io/z9f4v/.

## Abbreviations

BCT: Behavior change technique
ISCED: International Standard Classification of Education
