# Supplementary material for: Effects of behavioral performance, intrinsic reward value, and context stability on the formation of a higher-order nutrition habit: an intensive longitudinal diary study
Source: Int J Behav Nutr Phys Act. 2022 Aug 12;19:105. doi: 10.1186/s12966-022-01343-8 (PMC9372943; doi:10.1186/s12966-022-01343-8)
Supplement: Supplementary file 1 — Additional file 1. Comparison of methodological characteristics of this study with the guidelines for tracking real-world habit formation by Gardner et al. (2022). [file 12966_2022_1343_MOESM1_ESM.pdf]

The application of the rigorous guidelines for habit formation (Gardner et al., 2022) to this study

Domain	Criteria	Implementation in this study
Design	D1. Habit formation studies must focus on the strengthening of one or more specific cue-behaviour associations	Target habit: higher-order habit of filling half of the plate with vegetables at dinner time
	D2. Habit formation studies must be conducted in settings in which there is reason to expect habit to meaningfully strengthen	Target habit is context-contingent (the context of having dinner), it can be performed on a regular basis, and not all individuals already engage in the target behavior
	D3. Analyses of potential moderators of habit formation must adopt designs sensitive to the temporal nature of relationships between repetition, habit development, and potential moderators	Usage of an intensive longitudinal design, investigation of lagged effects; habit can be performed once per day → assessment of measures once per day
	D4. Habit formation studies should use longitudinal designs	Usage of an intensive longitudinal design with 56 subsequent days as points of measurement
Measurement	M1. Habit formation studies must attempt to measure habit	Application of the self-report behavioral automaticity index (SRBAI) to measure habit strength
	M2. Habit formation studies must not infer habit from behavioural frequency alone	Distinct measurement of behavioral performance and habit strength
	M3. Habit formation studies must measure habit in relation to a behaviour of interest	Framing of the items in the SRBAI related to the target behavior, i.e., "filling half of the plate with vegetables at dinner is something…"
	M4. Habit formation studies should use habit measures relating to behaviour at an appropriate level of specificity	Application of the higher-order habit of filling half of the plate with vegetables at dinner time as a specific behavior (instigation habit), however, the higher-order nature allows variety in the behavioral execution and contributes to a varied diet.
	M5. Habit formation studies should use context-specific habit measures	Context specificity or stability was explicitly examined as a potential moderator for habit formation.

		Context stability of dinner (regarding time, location, social environment, types of vegetables and activities prior to and post dinner being “as usual”) was assessed via six items on a 7-point Likert-scale (1 = ‘do not agree at all’ to 7 = ‘totally agree’)
	M6. Habit formation studies should measure habit over multiple timepoints	Usage of an intensive longitudinal design with 56 subsequent days as points of measurement
Analysis and Interpretation	AI1. Habit formation studies must acknowledge habit strength as a continuum	Modelling of habit strength as a continuous measure in a multilevel regression, addition of an autoregressive effect of habit strength (i.e., habit strength at t1-1) to all models
	AI2. Habit formation tracking studies should not infer effects of repetition from measures of time	Explicit assessment of behavioral performance with the dichotomous item “Did you fill half of your plate with vegetables at dinner?” (0 = no, 1 = yes).
	AI3. Habit formation tracking studies must not assume that habit develops linearly	addition of an autoregressive effect of habit strength (i.e., habit strength at t1-1) to all models
	AI4. Habit formation tracking studies must involve analyses that account for individual differences in the growth trajectory	Application of multilevel modelling with daily diaries (level 1) being nested within individuals (level 2), including a random slope for time; Explicit empirical examination of moderators of habit growth over time
