# Supplementary material for: Effects of behavioral performance, intrinsic reward value, and context stability on the formation of a higher-order nutrition habit: an intensive longitudinal diary study
Source: Int J Behav Nutr Phys Act. 2022 Aug 12;19:105. doi: 10.1186/s12966-022-01343-8 (PMC9372943; doi:10.1186/s12966-022-01343-8)
Supplement: Supplementary file 2 — Additional file 2. STROBE checklist. Checklist which references to the pages in the manuscript which contain the essential descriptions of the study. [file 12966_2022_1343_MOESM2_ESM.pdf]

STROBE Statement—Checklist of items that should be included in reports of ***cross-sectional studies***

	Item No	Recommendation
**Title and abstract**	1	**(*a*) Indicate the study's design with a commonly used term in the title or the abstract:** *p. 1:"Effects of behavioral execution, reward value, and context stability on the formation of a higher-order nutrition habit: An intensive longitudinal diary study"*
		**(*b*) Provide in the abstract an informative and balanced summary of what was done and what was found:** *Methods: Participants were recruited via mailing lists and posts on social media platforms. N = 199 participants (Mage = 37.10 years, SD = 13.00, 86.93% female) received an online intervention for building the higher-order habit of filling half of their plates with vegetables at dinner and completed one daily online survey for up to 56 days, including the assessment of habit strength, behavioral performance, intrinsic reward value, and context stability, providing a total of N = 6352 daily measurements. N = 189 participants (N = 4175 measurements) could be included in the primary analysis. Utilizing multilevel modeling, we analyzed the impact of behavioral performance, intrinsic reward value, and context stability, as well as their interaction effects, on habit strength on the next day.*<br>*Results: Habit strength significantly increased over time. This effect was strengthened in persons with high mean levels of behavioral performance. Furthermore, mean levels of behavioral performance, intrinsic reward value, and context stability were all positively related to mean levels of habit strength. There were no positive effects of daily intraindividual variations in the three examined factors on habit strength at the next day. There was an unexpected negative effect of daily behavioral performance on habit strength at the next day. We found little to no evidence for our expected and pre-registered interaction effects. In an additional exploratory analysis, there were positive effects of daily intraindividual variations in the three factors on habit strength at the same day.*<br>*Conclusions: We found that behavioral performance, intrinsic reward value, and context stability were all independent predictors of habit strength of a higher-order habit at the between-person level. However, we did not find the expected associations at the within-person level. Habit interventions should promote the consistent performance of the target behaviors in stable contexts."*
**Introduction**		
Background/rationale	2	**Explain the scientific background and rationale for the investigation being reported:**<br>*p. 5-7:"**The habit formation process***<br>*To increase the strength of a certain habit, the specific behavior must be repeatedly performed in the same context until a mental association between the context and the behavior is established [23]. Habit strength typically increases in the shape of an asymptotic curve with fast increases in the beginning, peak values being reached after around two months, and with large variability between individuals [24,25]. Intensive longitudinal designs can help to disentangle differential effects in the habit formation process [24,26,27].".*<br>***Determinants of habit formation***<br>*Gardner and Lally [28,29] have proposed a framework for habit formation, which includes four stages: Intention formation (stage 1), action initiation (stage 2),*

		behavioral repetition (stage 3a), and development of a cue-behavior-association (stage 3b). Each of these stages can be a potential starting point for examining and modifying factors which may promote successful habit formation. For example, goal setting as a behavior change technique (BCT; BCT 1.4.) [30] could be used to support intention formation (stage 1), whereas action planning (BCT 1.4.) in the form of implementation intentions [31] could be used to promote action initiation (stage 2) and behavioral repetition (stage 3a). Behavioral repetition could be further supported by self-monitoring of behavior (BCT 2.3.), and the development of cue-behavior-associations could be quickened by using rewards (BCT 10.3.), see also [9].<br>*In this study, we simultaneously examine the independent influence of behavioral performance, intrinsic reward value related to vegetable consumption, and the stability of the context in which the target behavior is enacted, on the formation of a higher-order nutrition habit. All three factors have been discussed on a theoretical level [8,23] and researched within the frameworks of different health behaviors, but mainly physical activity, and mainly on the between-person level (i.e., by examining the influence of the factors on interindividual differences in habit formation over time) [21,32–37]. With regard to dietary behavior, positive associations between consistent behavioral performance and habit strength [21,25,38,39] and mixed associations between intrinsic reward value and habit strength exist [21,32,39]. Repeated behavioral performance clearly relates to stage 3a and 3b of the habit formation framework [28] and should promote successful habit formation. The intrinsic reward value related to vegetable consumption could potentially enhance motivation or performance and accelerate cue-behavior learning and therefore positively affect multiple stages of the habit formation process [28,40]. With regard to the third factor, context stability, some studies have investigated which kind of cues (e.g., event-based vs. time-based cues) promote habit formation best [24,41]. However, there is no research on the influence of the stability of contextual cues on the formation of real-world nutrition habits [36]. Regarding vegetable consumption at dinnertime, there are various contextual factors such as the time and position in the daily routine (e.g., after work or exercising), location, social context (i.e., how many other persons were present), and the type of vegetables consumed."*
Objectives	3	**State specific objectives, including any prespecified hypotheses:**<br>*p. 7-8: "**Aim of the study***<br>*Following design and measurement guidelines for tracking real-world habit formation [27], this study examines both between- (person level) and within-person (day level) effects of behavioral performance, intrinsic reward value, and context stability as determinants of habit formation of a higher-order habit of 'filling half the plate with vegetables at dinner time' [21] following a short habit intervention in an intensive longitudinal design. With this study, we adhere to the guidelines for rigorous habit formation research [42]. Additional file 1 contains the methodological characteristics of this study against the criteria proposed by Gardner et al. [42].*<br>*We expect 1) a positive effect of time since the intervention on habit strength at the within-person level, that is, behavioral automaticity will increase over time; 2) positive between-person effects of consistent behavioral performance, context stability, and intrinsic reward value as well as positive two-way interaction effects of these predictors with time on habit strength; 3) positive effects of behavioral performance, context stability, and intrinsic reward value as well as the two-way interactions with behavioral performance on habit strength at the within-person level.*

**Methods**		
Study design	4	**Present key elements of study design early in the paper:**<br>*p. 7-8: Following design and measurement guidelines for tracking real-world habit formation [27], this study examines both between- (person level) and within-person (day level) effects of behavioral performance, intrinsic reward value, and context stability as determinants of habit formation of a higher-order habit of 'filling half the plate with vegetables at dinner time' [21] following a short habit intervention in an intensive longitudinal design.*<br>and p. 9: "*After registration and provision of informed consent, participants received a link to the baseline assessment where participants provided demographic information, information about control variables, as well as variables of interest. Subsequently, they completed a single session habit formation intervention (see below). The diary period started the day after participants completed the baseline assessment; participants received one daily survey for eight weeks (i.e., 56 days) at 8.30 p.m. and were asked to fill out the daily survey after having eaten dinner. Completion of the survey was possible until 11 p.m."*
Setting	5	**Describe the setting, locations, and relevant dates, including periods of recruitment, exposure, follow-up, and data collection:**<br>*p. 9:"Participants were recruited via mailing lists, posts on social media platforms, and paid advertisement via social media platforms. Data collection for the baseline assessment took place between July 2020 and January 2021. Participants were eligible if they were at least 18 years old, had access to a computer or mobile device and email, as well as a WhatsApp account. Participants had the opportunity to enter a lottery to win one out of ten online shopping vouchers (worth 50€ each). The winning probability was linked to participants' study participation, in that the winning probability increased with every completed survey and photo documentation of their dinner. After registration and provision of informed consent, participants received a link to the baseline assessment where participants provided demographic information, information about control variables, as well as variables of interest. Subsequently, they completed a single session habit formation intervention (see below). The diary period started the day after participants completed the baseline assessment; participants received one daily survey for eight weeks (i.e., 56 days) at 8.30 p.m. and were asked to fill out the daily survey after having eaten dinner. Completion of the survey was possible until 11 p.m."*
Participants	6	**(*a*) Give the eligibility criteria, and the sources and methods of selection of participants**<br>Participants were eligible if they were at least 18 years old.
Variables	7	**Clearly define all outcomes, exposures, predictors, potential confounders, and effect modifiers. Give diagnostic criteria, if applicable**<br>The outcome and the predictors are clearly described in the methods section.
Data sources/ measurement	8*	**For each variable of interest, give sources of data and details of methods of assessment (measurement). Describe comparability of assessment methods if there is more than one group**<br>p. 10-12: "**Measures**<br>***Baseline assessment***<br>*Demographic variables of age (years), weight (kg), height (cm), current state of employment, and highest educational attainment were assessed at baseline. Educational level was coded according to the International Standard Classification of*

Education (ISCED) [45] and the recommendations by Eurostat [46] into the levels low (up to completing lower secondary education; ISCED levels 0-2), medium (completing higher secondary education and post-secondary, non-tertiary education; ISCED levels 3-4) and high (from completing the first stage of tertiary education up to completing doctoral level; ISCED levels 5-8), which allows cross-country comparisons [46].

Nutrition preferences (e.g., omnivore, vegetarian, vegan) were assessed with the self-generated item "What most closely reflects your current diet?". Participants could choose one out of six options (omnivore, vegetarian, vegan, pescatarian, paleolithic, or other).

Food intolerances were assessed with the self-generated item "Do you have any food intolerances?" (no = 0, yes = 1).

Average vegetable intake at dinner and average daily vegetable intake was assessed in 0.25-portion steps with two adapted dropdown items [47]; example item: 'Please indicate how many servings of vegetables you have consumed per day, on average, over the past seven days' after receiving information about portion sizes, based on the recommendation of the German Federal Ministry of Nutrition and the German Five-A-Day Campaign [48,49]

Health consciousness was assessed with five items [50] on a 5-point Likert-scale (1 = 'do not agree at all' to 5 = 'totally agree'); example item: "Living life in best possible health is very important to me"; Cronbach α = 78. For the analyses, the mean value of the five items was used.

The weekly frequency of eating dinner was assessed with the self-generated item "How many days per week do you eat dinner?" on an 8-point Likert-scale (0 = '0 days' to 7 = '7 days').

The context stability of the dinner (stability of dinner time, location, social environment, types of vegetables, and activities prior to and post dinner) was assessed with six items on an adapted scale by Pimm et al. (2016) on a visual analogue scale (0% = 'always changing' to 100% = 'always the same'); example item: "How consistent is the location at which you eat dinner?"; Cronbach α = 74. For the analyses, the mean value of the six items was used and divided by 100.

Daily survey

Habit strength was assessed with the Self-Report Behavioral Automaticity Index [14]. After the prefix "filling half of the plate with vegetables at dinner time is something…", participants were asked to rate four statements on behavioral automaticity, e.g., "…I do automatically" on a 7-point Likert-scale (1 = 'do not agree at all' to 7 = 'totally agree'). For the analyses, the mean value of the four items was used. Internal consistency, assessed with Nested Alpha for multilevel data [51] was α = .99.

Behavioral performance was assessed with the dichotomous item "Did you fill half of your plate with vegetables at dinner?" (0 = no, 1 = yes).

The intrinsic reward value of dinner was assessed via two self-generated items with a 5-point Likert-scale (1 = 'do not agree at all' to 5 = 'totally agree') regarding the tastiness of the dinner; example item: "The vegetables at dinner were tasty"; internal consistency, assessed with Nested Alpha for multilevel data [51], was α = 1.00.

Context stability of dinner (regarding time, location, social environment, types of vegetables and activities prior to and post dinner being "as usual") was assessed via six adapted items [33] on a 7-point Likert-scale (1 = 'do not agree at all' to 7 =

		'totally agree'); example item: "Dinner tonight took place in the same location as usual.". For the analyses, the mean value of the six items was used. Internal consistency, assessed with Nested Alpha for multilevel data [51], was α = .94".
Bias	9	**Describe any efforts to address potential sources of bias**<br>See item #8.
Study size	10	**Explain how the study size was arrived at**<br>Study recruitment stopped after the last paid social media advertisement campaign ended. We estimated to need at least 80-90 participants with about 3570 data points:<br>p. 8: *"We aimed to recruit about 80-90 participants with about 3570 data points (85 participants on level 2 * 56 daily surveys on level 1) to reliably estimate small fixed effects at the day-level, in case of small ICCs, as well as at least medium sized cross-level interaction effects with medium slope variances (31,32)."*
Quantitative variables	11	**Explain how quantitative variables were handled in the analyses. If applicable, describe which groupings were chosen and why**<br>p. 12: *"For examining between-person effects, values of the predictors represented the individual person-mean (43). For within-person analyses, predictors were centered within individuals such that positive values of predictors represented higher than average values, compared to the individual person-means, i.e., daily deviations from the person mean (43)."*
Statistical methods	12	***(a) Describe all statistical methods, including those used to control for confounding:***<br>p. 12*:"To examine the simultaneous influence of behavioral performance, context stability, and intrinsic reward value on habit strength at the next day, as well as habit formation over time, we specified two multilevel models using the R-package lmerTest [52]. Due to the robustness of multilevel models to deviations from normal distribution [53], we did not alter primary data in any way. In all multilevel models, daily diaries (level 1) were nested within individuals (level 2). For examining between-person effects, values of the predictors represented the individual person-mean [54]. For within-person analyses, predictors were centered within individuals such that positive values of predictors represented higher than average values, compared to the individual person-means, i.e., daily deviations from the person mean [54]. In the whole manuscript, we report the standardized effects.*<br>*We conducted the pre-registered analyses with all participants (model 1a) as well as all participants who responded to more than 50% of the daily surveys (high compliance sample; model 1b) to test the robustness of the results. The models included a random intercept for persons, person-centered values and person-specific means of behavioral performance, context stability, and intrinsic reward value at t1, and an autoregressive effect of habit strength (i.e., habit strength at t1-1) as independent variables. As dependent variables, habit strength at t1+1 were analyzed. All models included time and log(time) at t1 as independent variables to take the asymptotic nature of the habit formation curve into account [24,25]. We specified random slopes for log(time) to model the between-person variability in habit formation over time and to examine cross-level interactions. The models further included the following interaction effects: 1) cross-level interactions of log(time) with person-specific means of behavioral performance, context stability, and intrinsic reward value to predict between-person variability in habit formation over time and 2) interactions of person-centered behavioral performance with person-centered context stability and intrinsic reward value. Additionally, we also exploratorily*

		estimated the comparable within-person effects on habit strength at the same day (models 2a and 2b; Additional file 4).”
		**(*b*) Describe any methods used to examine subgroups and interactions**:<br>Not applicable.
		**(c) Explain how missing data were addressed**<br>There were no variable-specific missings at the day-level, that is, when participants filled out a daily survey, they reported on all variables. When participants did not complete all daily surveys, we still included them in the analyses because mixed models can handle missing observations at the lower level (daily surveys).
		**(*d*) If applicable, describe analytical methods taking account of sampling strategy**<br>We applied multilevel modelling (see item #12) because the daily surveys were clustered within participants.
		**(*e*) Describe any sensitivity analyses**<br>p. 12: “*We conducted the pre-registered analyses with all participants (model 1a) as well as all participants who responded to more than 50% of the daily surveys (high compliance sample; model 1b) to test the robustness of the results.”*
**Results**		
Participants	13*	**(a) Report numbers of individuals at each stage of study—eg numbers potentially eligible, examined for eligibility, confirmed eligible, included in the study, completing follow-up, and analysed**<br>See Fig. 1/ Flow chart.
		**(b) Give reasons for non-participation at each stage**<br>See Fig. 1/ Flow chart.
		**(c) Consider use of a flow diagram**<br>See Fig. 1/ Flow chart.
Descriptive data	14*	**(a) Give characteristics of study participants (eg demographic, clinical, social) and information on exposures and potential confounders:**<br>Baseline characteristics are provided in Table 1.
		**(b) Indicate number of participants with missing data for each variable of interest:**<br>The number of participants with missing data at the day-level is provided in the participant flow chart (Fig. 1).
Outcome data	15*	**Report numbers of outcome events or summary measures**<br>See Table 2.
Main results	16	***(c)* Give unadjusted estimates and, if applicable, confounder-adjusted estimates and their precision (eg, 95% confidence interval). Make clear which confounders were adjusted for and why they were included**<br>The full models with unadjusted standardized estimates and 95% CI are provided in Table 3 and Additional file 4.
		**(*b*) Report category boundaries when continuous variables were categorized**<br>Not applicable.
		***(c)* If relevant, consider translating estimates of relative risk into absolute risk for a meaningful time period**<br>Not applicable.
Other analyses	17	**Report other analyses done—eg analyses of subgroups and interactions, and sensitivity analyses**<br>The results of the robustness checks are provided on p. 15: ***“Analyses with high***

		compliance sample. *In the model with the high compliance sample (model 1b), all statistically significant main and interaction effects of model 1a replicated (see Table 3).”*
**Discussion**		
Key results	18	**Summarise key results with reference to study objectives**<br>We summarise key results on p. 16: *“First, we found the expected positive effect of time on habit strength. Second, regarding the between-person level effects, we found that mean levels of behavioral performance, intrinsic reward value, and context stability all were independent predictors of mean habit strength levels. Regarding the size of the effects, context stability showed the strongest associations, directly followed by behavioral performance, whereas the association intrinsic of reward value was significantly smaller. However, when including the interaction effects with time, we only found the expected interaction effect with behavioral performance, but not with intrinsic reward value or context stability. There were none of the expected within-person effects of daily variations in behavioral performance, intrinsic reward value, and context stability with habit strength at the next day (but there were positive effects in comparable exploratory analyses with habit strength at the same day as dependent variable). In the following, we will discuss the findings of each of our three proposed predictors separately across the between- and within-person level.”* We further integrate the results in the existing literature (see p. 16-19).
Limitations	19	**Discuss limitations of the study, taking into account sources of potential bias or imprecision. Discuss both direction and magnitude of any potential bias**<br>p. 18-19: *“On the other hand, the present study also has some limitations. First, we integrated the stability of six contextual variables into one measure of context stability. Disentangling which factor shows the strongest association with habit strength would have exceeded the scope of our study, however recent research suggests there may be differences in the relevance of different cues for habit formation [36]. To provide practical guidelines on habit formation, it would be important to understand which contextual factors are most helpful for habit formation [59]. Second, we examined the formation of a pre-set higher-order habit which might not reflect participants’ own goals or fit to their daily schedules. Although this study was rather about the processes behind habit formation than intervention delivery, it might be that these processes unfold in a different manner when participants receive an intervention which is better tailored to their needs. Third, we used self-report measures which can induce common method bias [56] and lead to biased recall. However, in research on dietary behavior [60] and habit formation [23], subjective measures are frequently used and might be the most appropriate and available tools at a large scale. Individuals often have the most accurate information regarding their behavior, subjective measures are low in cost, and easily applicable in online studies [60]. Furthermore, some measures such as habit strength regarding real-world habits may not be objectively assessable [23]. We aimed to reduce recall bias by assessing the variables of interest in timely proximity to the completion of the target habit. We also partially reduced same source bias, which can exaggerate common method bias, by examining lagged effects [57]. Nevertheless, future research can benefit from using*

		more objective measures where possible such as automatic coding of food pictures to capture behavioral performance [61] or using neurophysiological markers such as heart rate or skin conductance for assessing affective responses to meals [62]. Finally, our study did not include a control group without habit formation intervention and thus does not allow conclusions regarding overall intervention effectiveness. However, as we aimed to examine important moderating factors that might contribute to successful habit formation instead of overall intervention effectiveness, a control group was not necessary."
Interpretation	20	**Give a cautious overall interpretation of results considering objectives, limitations, multiplicity of analyses, results from similar studies, and other relevant evidence**<br>p. 22: *"The fact that our study provides additional proof for habit formation using a higher-order habit could be helpful for future habit-based interventions, especially in complex health behaviors such as nutrition. In real-world settings, higher-order food habit such as filling half of the plate with vegetables at dinner time could promote a high variety in vegetable choice which may improve health outcomes, but findings are mixed [67–70]. In addition, individuals could maintain interest in the behavior over time and be creative and intrinsically motivated in the composition of different types of vegetables. Considering global and national dietary recommendations, food-based dietary guidelines should include specific and actionable advice on the consumption of healthy food groups such as fruits and vegetables. Our results and the results of others [21] show that behavioral targets such as filling half of the plate with fruits and/or vegetables, which is already recommended in some dietary guidelines [71], are suitable for habit formation and thereby the promotion of a healthy diet. Behavioral performance was revealed to be the strongest predictor for habit strength, followed by context stability. Intervention designers and practitioners should thus focus on fostering the consistent performance of the target behavior – ideally in stable reoccurring contexts – for establishing strong healthy eating habits. To ensure context-dependent repetition, additional self-regulatory strategies may further support habit formation [29]. For example, planning with implementation intentions, or self-monitoring, which we included in our intervention, are frequently utilized behavior change techniques in habit-based intervention [9]. Furthermore, other additional behavior change techniques can support habit formation in each of the three phases of habit formation [28,29]. For example, increasing the intrinsic reward value by making the target behavior more enjoyable [72] could further strengthen habit formation."* And p. 22-23: *"This study examined the effects of behavioral performance, intrinsic reward value, and context stability on habit strength of a higher-order nutrition habit following a short habit formation intervention. Behavioral performance, intrinsic reward value, and context stability did not predict habit strength at the next day (but independently predicted habit strength at the same day). Future studies could investigate whether time lag intervals other than one day better capture the effects of behavioral performance, intrinsic reward value, and context stability on habit strength, and disentangle which contextual stability determinants (cues) are most influential. Consistent behavioral performance and context stability further emerged as key predictors of habit strength on the between-person level. Thus, in practice, intervention designers should focus on supporting individuals to perform the target habit frequently in a stable context by combining*

		different behavior change techniques that might promote these factors (e.g., implementation intentions for increasing behavioral performance).”
Generalisability	21	**Discuss the generalisability (external validity) of the study results**<br>We expect the results to be generalizable because we examined basic habit formation mechanisms which should apply in every habit formation and our sample was relatively diverse regarding age and educational level.
**Other information**		
Funding	22	**Give the source of funding and the role of the funders for the present study and, if applicable, for the original study on which the present article is based**<br>p. 21: “*This work was supported by the Subdivision Health Psychology of the German Psychological Society (DGPs) within the Peer-Mentoring Grant. The funders had no role in study concept and design, data collection, analysis and interpretation, or the preparation and the decision to submit this manuscript for publication. The publication of this article was funded by the University of Mannheim”*

*Give information separately for exposed and unexposed groups.

Note: An Explanation and Elaboration article discusses each checklist item and gives methodological background and published examples of transparent reporting. The STROBE checklist is best used in conjunction with this article (freely available on the Web sites of PLoS Medicine at http://www.plosmedicine.org/, Annals of Internal Medicine at http://www.annals.org/, and Epidemiology at http://www.epidem.com/). Information on the STROBE Initiative is available at www.strobe-statement.org.
