# Supplementary material for: Effects of behavioral performance, intrinsic reward value, and context stability on the formation of a higher-order nutrition habit: an intensive longitudinal diary study
Source: Int J Behav Nutr Phys Act. 2022 Aug 12;19:105. doi: 10.1186/s12966-022-01343-8 (PMC9372943; doi:10.1186/s12966-022-01343-8)
Supplement: Supplementary file 4 — Additional file 4. Exploratory analysis of same-day associations. [file 12966_2022_1343_MOESM4_ESM.pdf]

Table 1 Results of the same-day multilevel models for the outcome habit strength with all participants (model 2a) and participants with high compliance (model 2b)

	Model 2a				Model 2b			
	**β**	***SE***	***95% CI***	***p***	**β**	***SE***	***95% CI***	***p***
Intercept	0.02	0.05	[-0.07, 0.11]	.647	0.01	0.06	[-0.10, 0.12]	.865
**Within-person effects**								
Autoregressive effect	0.18	0.01	[0.15, 0.20]	**< .001**	0.18	0.01	[0.15, 0.20]	**< .001**
Time	-0.00	0.01	[-0.03, 0.03]	.841	0.00	0.01	[-0.03, 0.03]	.853
log(time)	0.16	0.02	[0.12, 0.20]	**< .001**	0.15	0.02	[0.10, 0.19]	**< .001**
Behavioral performance (cwc)	0.10	0.01	[0.09, 0.12]	**< .001**	0.10	0.01	[0.09, 0.11]	**< .001**
Intrinsic reward value (cwc)	0.04	0.01	[0.03, 0.05]	**< .001**	0.03	0.01	[0.02, 0.05]	**< .001**
Context stability (cwc)	0.02	0.01	[0.01, 0.03]	**.002**	0.01	0.01	[0.00, 0.02]	**.021**
Behavioral performance (cwc)*Intrinsic reward value (cwc)	-0.02	0.00	[-0.03, -0.01]	**< .001**	-0.02	0.00	[-0.03, -0.01]	**< .001**
Behavioral performance (cwc)*Context stability (cwc)	-0.01	0.00	[-0.02, 0.00]	.294	-0.01	0.01	[-0.02, 0.00]	.262
**Between-person effects**								
Behavioral performance (M)	0.19	0.04	[0.10, 0.27]	**< .001**	0.12	0.06	[0.01, 0.23]	**< .040**

Intrinsic reward value (M)	0.13	0.05	[0.04, 0.21]	**.006**	0.18	0.06	[0.06, 0.30]	**.003**
Context stability (M)	0.20	0.05	[0.11, 0.30]	**< .001**	0.28	0.06	[0.16, 0.39]	**< .001**
log(time)*Behavioral performance (M)	0.04	0.02	[0.01, 0.07]	**.015**	0.04	0.02	[0.00, 0.07]	.061
log(time)*Intrinsic reward value (M)	0.03	0.02	[-0.01, 0.06]	.137	0.03	0.02	[-0.01, 0.07]	.087
log(time)*Context stability (M)	0.01	0.02	[-0.02, 0.05]	.541	0.01	0.02	[-0.03, 0.05]	.591
**Random effects**								
SD(Residual)	0.33	0.01	[0.32, 0.34]		0.32	0.01	[0.32, 0.33]	
SD(Intercept)	0.61	0.03	[0.54, 0.68]		0.60	0.04	[0.53, 0.68]	
SD(Slope (log(time)))	0.20	0.01	[0.17, 0.22]		0.19	0.01	[0.16, 0.22]	
ICC	0.79				0.79			
N	189				121			
Observations	4135				3751			
Marginal R^2 / Conditional R^2	0.354 / 0.864				0.394 / 0.873			

Note. The same-day models were estimated with habit strength measured at the same day as the predictors. (M) = Person-mean values. (cwc) = Values centered within clusters (persons). ICC = Intra-class correlation. *N* = Number of participants. *p*-values < .050 are marked in bold. Model 2a contains analyses with all participants that could be included in the analyses. Model 2b contains analyses with participants who responded to more than 50% of the daily surveys (high compliance sample).
